# Comparison of Adjuvant Hypertonic Saline and Normal Saline for Epidural Block in Patients with Postherpetic Neuralgia: A Double-Blind, Randomized Trial

**DOI:** 10.1155/2022/8081443

**Published:** 2022-11-21

**Authors:** Hyun-Jung Kwon, Doo-Hwan Kim, Seong-Sik Cho, Bokyoung Jeon, Myong-Hwan Karm, Seong-Soo Choi

**Affiliations:** ^1^Department of Anesthesiology and Pain Medicine, Asan Medical Center, University of Ulsan College of Medicine, Seoul 05505, Republic of Korea; ^2^Department of Occupational and Environmental Medicine, College of Medicine, Dong-A University, Busan 49201, Republic of Korea; ^3^Department of Dental Anesthesiology and Dental Research Institute, School of Dentistry, Seoul National University, Seoul 03080, Republic of Korea

## Abstract

**Background:**

In patients with postherpetic neuralgia (PHN), the effectiveness of epidural block and the benefits of adjuvant hypertonic saline (HS) have not been fully determined. Therefore, we investigated these issues in this study.

**Methods:**

At a tertiary medical center's single pain clinic in Seoul, Republic of Korea, patients complaining of PHN even after 4 months of herpes zoster onset were enrolled and randomly assigned to either the HS or normal saline (NS) group. After epidural block with adjuvant HS or NS administration according to each protocol, outcomes were assessed at baseline and one and three months after the intervention. The primary outcome was pain intensity on the numerical rating scale (NRS). The secondary outcomes were the insomnia severity index (ISI), the medication quantification scale (MQS), and the global perceived effect of satisfaction (GPES).

**Results:**

Thirty-six patients (NS: 17, HS: 19) were included in the intention-to-treat analysis. The estimated pain intensity decreased in both groups at one and three months after the procedure (*P* < 0.001), without a significant group difference. The estimated ISI and MQS were not significantly different at 1 month compared with baseline but significantly decreased at 3 months in each group (*P* < 0.001 and *P* < 0.001, respectively), without group differences. In addition, there was no difference between the groups on the GPES scale at one and three months after the procedure.

**Conclusions:**

Epidural steroid injection may have the advantages of short-term pain relief, improved sleep quality, and decreased medication usage in patients with PHN. In addition, adjuvant HS administration with epidural steroid injection did not show beneficial effects in patients with PHN. Further studies are needed to clarify the potential effectiveness of HS in treating neuropathic pain such as PHN. This trial is registered with KCT0002845.

## 1. Introduction

As a sequelae of acute herpes zoster (AHZ) in sensory neurons, postherpetic neuralgia (PHN) often lasts from several years to a lifetime. The incidence of PHN is 7%–50% at 3 months and 5%–32% at 6 months after AHZ [1]. These patients often suffer from severely agonizing pain that leads to sleep disturbance, impaired social activities, and decreased quality of life [1–3].

Although several pharmacologic treatment options for PHN, such as anticonvulsants, antidepressants, opioids, topical lidocaine, and topical capsaicin, can be used, they are sometimes insufficient to control the pain. In this case, interventional treatments such as epidural or intrathecal block with or without steroid injection, spinal cord stimulation, and sympathetic block can be considered [2, 4, 5]. The most popular interventional method for treating PHN was epidural blocks. However, this procedure may not be helpful for long-term pain relief in patients with PHN [2, 3]. In addition, the effectiveness of epidural blocks for PHN has not been fully determined [6].

In general, the analgesic effect of epidural injection with local anesthetics and steroids is mediated by inhibiting the synthesis of inflammatory mediators and decreasing neural excitability. Previously, it has been shown that the administration of adjuvant hypertonic saline (HS) for epidural block has the benefits of decreasing pain intensity and extending the duration of pain relief in patients with other types of chronic pain, such as pain due to spinal stenosis [7–9]. Although the mechanism of action of HS for epidural block is not yet fully clarified, high concentrations of sodium and chloride ions may play a role in blocking noxious stimuli with a neuromodulatory effect as well as in reducing nerve edema [7, 8]. These effects of HS may be reasonably applicable to patients with PHN.

Therefore, the present study aimed to investigate the effectiveness of adjuvant HS administration for epidural block in patients with PHN.

## 2. Materials and Methods

### 2.1. Participants and Study Design

This study was a randomized, double-blind, active-controlled clinical trial. The Institutional Review Board of our institution gave its consent for this study to be conducted (IRB approval number, 2017-0433). Each patient who took part in this trial provided their written, informed permission. Participants' confidentiality and privacy were upheld in every way. In addition, this study was performed in compliance with the Helsinki Declaration and filed with the Clinical Research Information Service (https://cris.nih.go.kr/KCT0002845) [10]. In reporting this study, we adhered to the CONSORT recommendations.

We evaluated the eligibility of PHN patients who visited our center's pain management clinic between May 2018 and December 2020. The following were the criteria for inclusion: (1) patients who complained of PHN even after 4 months of herpes zoster onset [11]; (2) patients (aged ≥18 years) who could understand and cooperate with the research purpose; (3) patients whose location of PHN was a dermatome below C2; (4) patients with a pain intensity ≥6 (out of 10) on the numerical rating scale (NRS); (5) patients who failed to control pain even when an effective dose of analgesics was administered; and (6) patients who voluntarily agreed in writing to participate in the present trial.

Exclusion criteria were as follows: (1) patients who refused to participate in this clinical trial; (2) patients whose PHN was located in the head or face; (3) patients who underwent any interventional procedure such as an epidural steroid injection, paravertebral block, pulsed radiofrequency, or spinal cord stimulation within 3 months; (4) patients who were pregnant or lactating; (5) patients who had hypersensitivity to local anesthetics, steroids, contrast dyes, or HS; (6) patients who had a coagulopathy; (7) patients with red flag signs (infection, cancer, fracture, and neurological deficits or cauda equine syndrome); and (8) patients with uncontrolled medical or psychiatric diseases, including opioid disuse.

### 2.2. Randomization and Blinding

Participated patients were randomly assigned to one of two groups: the HS group (administered an epidural steroid injection with HS) or the normal saline (NS) group (administered an epidural steroid injection with NS). Based on a computer-generated randomization method, an impartial data manager divided the patients among the groups. Each patient's randomization number was concealed from both the trial participants and the outcome assessor, an independent pain specialist. From the time of the procedure to the follow-up period, the interventional pain physicians remained unaware of which group the patients belonged to.

### 2.3. Intervention: Epidural Steroid Injection with HS or NS

Two pain physicians with more than ten years of experience conducted every procedure in this study. No premedication or sedatives were used in all participants. In patients selected according to the inclusion criteria, the target site of injection was the epidural space of the nerve affected by herpes zoster, through the parasagittal approach. All epidural steroid injection procedures were performed under fluoroscopic guidance. All epidural blocks were performed under a fluoroscopy system (OEC 9800, GE Healthcare, Little Chalfont, UK). During the procedure, each patient was placed in the prone position, and vital signs (pulse oximetry, blood pressure, and electrocardiography) were monitored. Sterile preparation and draping of the injection area were performed. Regarding the parasagittal interlaminar approach, the anteroposterior (AP) radiographic view was obtained to confirm the targeted vertebral level. Then, the image intensifier was cephaladly or caudally tilted to make the targeted interlaminar space more visible. After deciding the appropriate needle insertion point, local infiltration with 2 mL of 1% lidocaine was performed on the skin and soft tissues. A 20-gauge Tuohy needle was gently advanced under guidance by fluoroscopy. After placing the needle tip between the laminae, being ipsilateral to the symptomatic side of the patient on AP view, the needle was gently advanced using the lateral or contralateral oblique view while confirming the depth by visualizing the spinolaminar line or ventral interlaminar line, respectively [12–14]. Upon feeling the ligamentous resistance, the stylet was removed. Then, the needle was further advanced in millimeter increments using the loss of resistance technique to reach the epidural space. In a case of cervical epidural block above the C6 level, an 18-gauge Tuohy needle was inserted at the C6-7 or C7-T1 level for an epidural catheter insertion to reach the target cervical level. After an aspiration test to exclude cerebrospinal fluid or blood, 0.5–2.0 mL of contrast dye (Omnipaque 300, GE Healthcare AS, Oslo, Norway) was injected under a real-time fluoroscopy guide to ensure sufficient dispersion to the epidural space and to avoid additional potential intrathecal or intravascular injection. Following verification of the radiographic imaging's proper needle placement and sufficient contrast distribution, 6 mL of 1% lidocaine was administered. Before administering the drugs, a nurse who was not related to this study opened the envelope containing the paper with the assigned group and filled the syringe with the drug according to the assignment in a place where the physician could not see it. Then, the syringe that was not discernible by color or volume was delivered to the physician. After 3 min of lidocaine injection, 3 mL of 10% HS containing 5 mg dexamethasone in the HS group or 3 mL of NS containing 5 mg dexamethasone in the NS group was slowly administered over 3 min. After the procedure, the administration of neuropathic medication and/or analgesics was allowed.

### 2.4. Outcome Assessments and Follow-Up

All study participants' baseline characteristics were gathered. Assessments of the procedure's outcome were made at the baseline, 1 month, and 3 months later. Before the procedure, all study participants were instructed on how to use the 11-point NRS (0 = no pain and 10 = worst possible pain) to assess the pain intensity of their PHN, along with the insomnia severity index (ISI), a 7-item self-report questionnaire with a total score ranging from 0 to 28, to rate their quality of sleep [15–17]. Furthermore, patient satisfaction and improvement were evaluated using the 7-point Likert scaled global perceived effect of satisfaction (GPES) [18]. In addition, analgesic alterations were accessed using the medicine quantification scale (MQS) III [19]. Adverse events that occurred throughout therapy and follow-up were documented.

The primary outcomes were the mean difference of NRS scores in pain intensity from baseline at one month and three months. The secondary outcomes included changes in ISI, MQS, and GPES and adverse events in each group over the course of the follow-up. At each follow-up evaluation, the differences from baseline in pain intensity, ISI, and MQS were assessed. If procedural complications occurred, they were recorded. Also, any adverse events were further assessed during the follow-up period.

All patients were informed of this guideline before participating in the trial. According to the patients' residual pain intensity at each follow-up, the prescription doses of each analgesic were either decreased or increased.

### 2.5. Sample Size

The study sample size was determined on the basis of a previous publication [8], in which the average degree of pain reduction 3 months after the procedure was estimated at 2.93 for the HS group, 1.52 for the NS group, and 1.6 for the standard deviation. Assuming a type 1 error of 0.05 (two-tailed) and a power of 0.80, a minimum number of 34 patients was required for between-group comparisons with the repeated analysis of variance test. By setting the dropout rate at 15%, we determined a total of 40 patients as the sample size.

### 2.6. Statistical Analysis

Absolute numbers and percentages are used to represent categorical variables. Continuous variables are displayed as medians with interquartile range or means with standard deviation or 95% confidence intervals. The categorical variables were evaluated by the *χ*^2^ test or Fisher's exact test appropriately. The Student's *t*-test or the Mann–Whitney *U* test was used to evaluate continuous variables, as applicable, to compare data between groups. All observed data were analyzed by intention-to-treat (ITT) analysis, despite loss to follow-up or dropout from the study. Given that data loss resulting from loss to follow-up was anticipated, a linear mixed-effect model was used to analyze and compare changes in continuous variables (NRS, ISI, MQS, and GPES) at baseline and one month and three months after the procedure within and between groups. SPSS version 21 (IBM Corp, Armonk, NY) and Stata version 13.1 (StataCorp LP, College Station, TX) were used for all data handling and statistical analysis. A statistically significant difference was determined to exist when the two-tailed *P* value was less than 0.05.

## 3. Results

**Figure 1:**
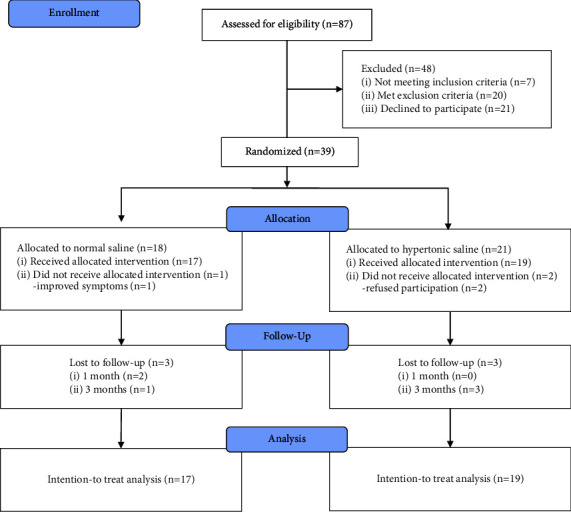
CONSORT (Consolidated Standards of Reporting Trials) flow diagram of participants in this trial.

Between May 2018 and December 2020, 88 consecutive patients diagnosed with PHN were screened for eligibility to be included in this study. A total of 40 patients who fulfilled both the exclusion and inclusion criteria agreed to participate in this study. Seven patients did not meet the inclusion criteria, 20 patients met the exclusion criteria, and 21 patients declined to participate. After randomization, 20 patients were assigned to the NS group and 20 patients were assigned to the HS group. Among the 20 eligible NS group patients, 1 patient did not receive the allocated intervention because of improved symptoms and 2 patients refused to participate after randomization. Among the 20 eligible HS group patients, 1 patient did not receive the allocated intervention because he refused to participate in the study. Finally, a total of 36 patients (17 in the NS group and 19 in the HS group) were included in the ITT analysis. All 36 patients were assessedat 1 at one and three months after the procedure, two NS group patients dropped out, whereas no HS group patient dropped out. At 3 months after the procedure, one NS group patient and three HS group patients dropped out (Figure 1). The reasons for dropout were as follows: five patients did not return for the follow-up visit, and one patient underwent another intervention before completing the study.

As shown in Table 1, the baseline demographic characteristics were not significantly different between the two groups. The most common site of lesions was the thoracic spine, without a group difference. As the primary outcome, the estimated mean changes in pain intensity (NRS) over the 3 months follow-up period are shown in Table 2. The results of the ITT analysis showed that the estimated pain intensity (NRS) in both groups was decreased at one and three months compared with baseline following each intervention (*P* < 0.001 and *P* < 0.001, respectively), without a group difference (*P* = 0.380). We did not find significant interactions in the NRS between groups and time (*P* = 0.942). Changes in ISI and MQS are shown in Tables 3 and 4, respectively. Although the estimated ISI and MQS were not different at 1 month compared with baseline, the ISI and MQS in both groups were improved at 3 months compared with baseline after each intervention (*P* < 0.001 and *P* < 0.001, respectively), without group differences (*P* = 0.123 and *P* = 0.899). No significant interactions in ISI and MQS were observed between groups and time (*P* = 0.981 and *P* = 0.465, respectively). In addition, the GPES scales at one and three months after the procedure were not significantly different between groups (Table 5).

We did not observe any serious complications during the injections. Several patients reported temporary injection pain during needling and injection, which was manageable and did not need any additional care. There were no other complications, such as intravenous injection, dural puncture, infection, or persistent neurologic impairment. Postprocedure complications were also not reported during the follow-up.

## 4. Discussion

The present study found that the intensity of pain was decreased by 3 months after epidural block in patients with PHN. However, the difference in pain intensity decrease between the HS and NS groups was not significant. The estimated ISI and MQS were also decreased3 at months without group differences.

Epidural nerve blocks are often performed in patients with PHN that are not well controlled by conservative treatment with neuropathic pain medication and/or analgesics. According to the report by Nahm et al., epidural nerve blocks were performed in 52.1% (interlaminar epidural injection in 32.7% and transforaminal epidural injection in 19.4%) of patients with PHN in the spinal area in Korea [6]. However, there are few data on the effectiveness or efficacy of epidural blocks in PHN, whereas the quality of evidence about relieving pain in AHZ and preventing PHN after AHZ is moderate [20, 21]. The speculation that nerve blocks, including epidural injections, might have some benefits for PHN was only based on some case series [20, 22]. In another previous report, satisfactory analgesia with more than 50% of global pain relief was achieved only in 20% of the 12 cases of epidural steroid injection at 1 week and 24 weeks after the procedures. In this report, the mean extent of pain relief was not presented as numbers like the NRS, but just as graphs [23]. A recent retrospective study reported that 45.2% of the patients with PHN had moderate-to-good pain relief at 3 months after epidural block [24]. Until now, the effectiveness of epidural injection in PHN has not been completely determined [2]. In the same context, an evidence-based guideline suggested that epidural steroid or anesthetic injection for the treatment of PHN is not supported by enough research [5]. A recent systematic review did not even mention epidural injection as an interventional treatment for PHN [25]. Nonetheless, in the present study, 30% pain relief was observed in the short-term period (3 months) after epidural injection in PHN, regardless of whether adjuvant HS was administered or not. Interestingly, administration of adjuvant HS helped in improving the sleep quality and decreasing the medication requirement of patients. To our knowledge, this study is unique in that it reported NRS differences, showing the extent of pain reduction in a randomized controlled study.

The pain relief achieved in both groups implies that epidural block with local anesthetics and steroids would be effective for PHN regardless of adjuvant HS administration. The sensory nerve affected by a neuropathic condition such as PHN has altered ion channel functioning and is usually sensitized with a lower threshold for action potentials. This produces disproportionate responses to stimuli and even spontaneous discharges. Local anesthetics block the generation and propagation of action potentials by inhibiting voltage-gated sodium channels. Generally, the affinity for ion channels in depolarized membranes is higher in neuropathic conditions [26]. The results of this study suggest that this mechanism may be applicable to PHN, similar to other neuropathic conditions. Meanwhile, corticosteroids are known to have roles in interrupting the inflammatory cascade, relieving edema of the affected nerves, and promoting nerve restoration [27]. However, epidural steroid injections may have more important roles in AHZ than in PHN, although the present results showed short-term effectiveness in managing PHN [28].

Epidural injection of HS was reported as an effective treatment in other neuropathic pain syndromes. In previous studies, epidural adhesiolysis with HS has shown favorable outcomes for short-term and long-term pain relief in patients with spinal stenosis, postspine surgery syndrome, and radiculopathy of any etiology, with moderate evidence [28–30]. Furthermore, a randomized controlled study by Shin et al. demonstrated the superiority of the addition of 10% HS to transforaminal epidural steroid injections (TFESI) for patients with chronic lumbosacral radicular pain caused by lumbar lateral canal stenosis [8]. The same research group retrospectively analyzed 246 patients with chronic low back pain associated with radicular pain and also showed that adding 10% HS to conventional TFESI provided a superior response over a 6-month period compared with conventional TFESI [7]. In light of the positive effects of HS reported earlier in other neuropathic pain syndromes, we hypothesized that epidural block with adjuvant HS may have merits for PHN, which is another specific group of neuropathic pain syndromes. According to the present results, adjuvant injection of 10% HS did not reduce pain intensity, improve sleep quality, or decrease drug utilization to a greater extent than conventional epidural block in patients with PHN.

As a conceivable reason, the chronicity of the disease might be a contributing factor. The mean total duration of pain was 26.6 (9.0–38.0) months in total participants, without substantial difference between the groups. According to the previous literature, nerve block in the acute stage of herpes zoster helps prevent PHN [28, 31]. Generally, it is accepted that the longer the duration of neuropathic pain, the higher the chance of central sensitization. Central sensitization is known as a crucial factor for poor response to any treatment [32–34]. In addition, the irreversible damage to the nervous system might be more extensive, involving the spinal cord and peripheral nerve endings from the beginning, which could also contribute to peripheral sensitization and hyperalgesia in PHN, unlike in spinal degenerative lesions investigated in prior studies. Considering that PHN does not originate from a mechanical spine problem but from damage to the nervous system itself, PHN caused by remnant nerve damage may be difficult to reverse or improve compared with neuropathic pain related to mechanical spine problems. Nevertheless, it is difficult to rule out that adjuvant HS administration with epidural steroid injection might relieve pain in patients with AHZ or early stage of PHN.

This pilot study has several limitations. First, the 16.7% follow-up loss rate with a small sample size may be criticized. Because this study was conducted in one of the largest hospitals in Korea, a considerable percentage of the patients came from various cities. The extent of follow-up loss may have been influenced by the high percentage of patients from remote areas. Although an ITT-based analysis and a linear mixed model were utilized to adjust for missing variables, the 3-month follow-up period in the present study may have been insufficient to evaluate the long-term clinical effectiveness of the epidural block. Therefore, it is necessary to further evaluate the long term treatment effect beyond 3 months. Second, the volume of injectate in the parasagittal interlaminar epidural injection in the present study was decided at 6 mL arbitrarily. Although the suggested volume of injectate for epidural injection varied in a few literature reports, the optimal volume is still to be determined [35–37]. However, a 6 mL volume in the parasagittal approach was considered sufficient to cover the targeted spinal nerve regardless of the spinal level [38]. Third, the protocol was confined to only a single bolus injection of HS. Since a single adjuvant injection is not necessarily the answer, this protocol can be modified to another, such as a several-day protocol with catheter insertion, which might affect the results in different ways [39]. Finally, the patients' initial emotional states could not be assessed. The perception of pain intensity and functional outcomes following the procedure may have been impacted by subjective emotions. However, considering that individuals with psychiatric or neurological disorders were not allowed to enroll in the trial, we think that emotional factors had a negligible impact on our findings.

## 5. Conclusions

Epidural steroid injection may have advantages of short-term pain relief, improved sleep quality, and decreased medication usage in patients with PHN. In addition, adjuvant HS administration with epidural steroid injection did not show beneficial effects in patients with PHN. Further studies are needed to clarify the potential effectiveness of HS in treating neuropathic pain such as PHN.

## Figures and Tables

**Table 1:** Baseline characteristics of the study participants.

	Normal saline (*N* = 17)	Hypertonic saline (*N* = 19)	*P* value
Age (years)	69.1 ± 10.1	65.7 ± 9.6	0.320
Sex (*n*, %)			0.739
Male	8 (47.1)	11 (57.9)
Female	9 (52.9)	8 (42.1)
Body mass index (kg/m^2^)	23.4 ± 4.1	24.2 ± 2.1	0.464
Diabetes (*n*, %)	4 (23.5)	4 (21.1)	0.858
Hypertension (*n*, %)	4 (23.5)	4 (21.1)	0.858
Immunocompromised state	7 (41.2)	6 (33.3)	0.733
Lesion site			0.989
Cervical	2 (11.8)	2 (10.5)
Thoracic	14 (82.4)	16 (84.2)
Lumbar	1 (5.9)	1 (5.3)
Number of levels	2.0 (1.0–2.0)	2.0 (1.0–2.0)	0.707
Duration of pain (months)	24.0 (9.0–33.0)	29.0 (9.0–38.0)	0.552
Basal pain intensity (NRS)	6.7 ± 1.7	6.4 ± 1.9	0.644
ISI (0–28)	14.3 ± 7.5	11.4 ± 5.1	0.184
MQS	11.7 ± 5.8	12.5 ± 7.1	0.756
Drug^a^			
Anticonvulsants	14 (82.4)	16 (84.2)	0.999
Antidepressants	8 (47.1)	11 (57.9)	0.739
Opioids	12 (70.6)	11 (57.9)	0.502
NSAIDs	1 (5.9)	2 (10.5)	0.999
Topical agents	2 (10.5)	1 (5.3)	0.593

Data are expressed as a number (%), mean ± standard deviation, or median (interquartile range). ^a^The individualized baseline drugs for managing pain were anticonvulsants (gabapentinoids and carbamazepine), antidepressants (tricyclic antidepressants and serotonin norepinephrine reuptake inhibitors), topical agents (transdermal lidocaine patches and capsaicin cream), and analgesics (opioids and NSAIDs). ISI, insomnia severity index; MQS, medication quantification scale; NRS, numerical rating scale; NSAIDs, nonsteroidal antiinflammatory drugs.

**Table 2:** Changes in the estimated pain scores after epidural block with or without hypertonic saline in patients with postherpetic neuralgia.

Variable	Time	Groups		Differences^a^	*P* value
		Normal saline (*N* = 17)	Hypertonic saline (*N* = 19)		
Pain (NRS)	Baseline	6.7 (5.8–7.6)	6.4 (5.6–7.2)	−0.3 (−1.5–0.9)	0.643
	1 month	5.2 (4.3–6.2)^b^	4.7 (3.9–5.6)^b^	−0.5 (−1.7–0.7)	0.425
	3 months	4.8 (3.9–5.8)^b^	4.3 (3.4–5.2)^b^	−0.5 (−1.8–0.8)	0.465

A numerical rating scale (NRS) was used to assess the intensity of postherpetic neuralgia pain. A mixed linear model was used in the statistical analysis. The data are presented as the estimated mean with a 95% confidence interval. The group difference was not significant (*P* = 0.380). The time effect was significant (*P* < 0.001). The *P* value of the interaction between the group and time was not significant (*P* = 0.942). ^a^Estimated difference in values between groups. ^b^*P* < 0.001 compared with the baseline in each group.

**Table 3:** Changes in the estimated insomnia severity index after epidural block with or without hypertonic saline in patients with postherpetic neuralgia.

Variable	Time	Groups		Differences^a^	*P* value
		Normal saline (*N* = 17)	Hypertonic saline (*N* = 19)		
ISI (0–28)	Baseline	14.3 (11.4–17.1)	11.4 (8.7–14.1)	−2.9 (−6.8–1.1)	0.152
	1 month	12.3 (9.4–15.3)	9.6 (6.9–12.3)	−2.7 (−6.7–1.3)	0.188
	3 months	11.5 (8.4–14.5)^b^	9.0 (6.1–11.8)^b^	−2.5 (−6.6–1.7)	0.238

The insomnia severity index (ISI) was used to assess insomnia. A mixed linear model was used in the statistical analysis. The data are presented as the estimated mean with a 95% confidence interval. The group difference was not significant (*P* = 0.123). The time effect was significant (*P* = 0.014). The *P* values of interaction between the group and time were not significant (*P* = 0.981). ^a^Estimated difference in values between groups. ^b^*P* < 0.001 compared with the baseline in each group.

**Table 4:** Changes in the estimated medication quantification scale after epidural block with or without hypertonic saline in patients with postherpetic neuralgia.

Variable	Time	Groups		Differences^a^	*P* value
		Normal saline (*N* = 17)	Hypertonic saline (*N* = 19)		
MQS	Baseline	11.8 (8.4–15.1)	12.5 (9.3–15.6)	0.7 (−3.9–5.3)	0.771
	1 month	9.9 (6.4–13.4)	10.9 (7.6–14.1)	1.0 (−3.8–5.8)	0.693
	3 months	7.4 (3.8–11.1)^b^	5.3 (1.9–8.7)^b^	−2.1 (−7.1–2.9)	0.402

The medication quantification scale (MQS) was used to assess the changes in medication usage. A mixed linear model was used in the statistical analysis. The data are presented as the estimated mean with a 95% confidence interval. The group difference was not significant (*P* = 0.899). The time effect was significant (*P* = 0.003). The *P* values of interaction between the group and time were significant (*P* = 0.465). ^a^Estimated difference in values between groups. ^b^*P* < 0.001 compared with the baseline in each group.

**Table 5:** Changes in the estimated global perceived effect of satisfaction after epidural block with or without hypertonic saline in patients with postherpetic neuralgia.

Variable	Time	Groups		Differences^a^	*P* value
		Normal saline (*N* = 17)	Hypertonic saline (*N* = 19)		
GPES	1 month	3.9 (3.2–4.6)	4.2 (3.6–4.8)	0.3 (−0.7–1.2)	0.559
	3 months	3.8 (3.1–4.5)	4.5 (3.8–5.1)	0.6 (−0.3–1.6)	0.186

The global perceived effect of satisfaction (GPES) was measured after epidural block in both groups. A mixed linear model was used in the statistical analysis. The data are presented as the estimated mean with a 95% confidence interval. The group difference was not significant (*P* = 0.657). The time effect was not significant (*P* = 0.272). The *P* values of interaction between the group and time were not significant (*P* = 0.495). ^a^Estimated difference in values between groups.

## Data Availability

The Institutional Review Board at Asan Medical Centre, Seoul, Korea, has put restrictions on the data used to support the study's conclusions in order to preserve patient privacy. For researchers who meet the requirements for access to secret data, the corresponding author will provide the data.
